# The Experience of Pleasure: A Perspective Between Neuroscience and Psychoanalysis

**DOI:** 10.3389/fnhum.2018.00359

**Published:** 2018-09-04

**Authors:** Lorenzo Moccia, Marianna Mazza, Marco Di Nicola, Luigi Janiri

**Affiliations:** ^1^Fondazione Policlinico Universitario Agostino Gemelli IRCCS, Università Cattolica del Sacro Cuore, Rome, Italy; ^2^Centro Psicoanalitico di Roma, Società Psicoanalitica Italiana, Rome, Italy

**Keywords:** pleasure, displeasure, pain, psychoanalysis, neuroscience, neuropsychoanalysis, affect, experience

## Abstract

Pleasure is more than a mere sensory event, but rather it can be conceptualized as a complex, multiform experience involving memory, motivation, homeostasis, and, sometimes, negative affects. According to Freud, affect is a perceptual modality that registers the internal drive state of the subject rather than the objective experience of the external world, and the quality of this perceptual modality is calibrated in degrees of pleasure and displeasure. Within this conceptual framework, the aim of drive is always pleasure, and objects become significant in so far as they provide a way of discharging drives pressure. Subsequent conceptual psychoanalytic developments have partially rejected such metapsychological theorizations, postulating that other intrinsic motivations that are independent from libido can be observed in humans. Intrinsic motivation broadly refers to a set of psychological concepts including the inherent propensity to pursue one’s choices, to seek out novelty and challenges, to satisfy curiosity and competence, and to extend one’s capacities and control over events. What these concepts have in common is an inner endorsement of one’s action, which is the sense that action is self-generated and is one’s own. The notions of pleasure, drives, and affects are all of utmost importance for a neuropsychoanalytic understanding of mental functioning, due to their capability to explain desire, thought, and behavior from the perspective of human subjective experience. The purpose of this paper is thus to discuss psychoanalytic conceptual developments that have addressed pleasure, drives, and affects, in the light of recent findings coming from neurosciences. In particular, we will explore for insights from Panksepp’s theory of primary-process emotional feelings, including the notion of “wanting” and “liking” as dissociable components of reward. In the last part of the paper, we will indicate possible theoretical implications for a neuropsychoanalytic understanding of libido-independent intrinsic motivations and their relationship with the self, including neuroscientific observations on self-related processes, agency, body-ownerships, and attachment.

## Pleasure and Affects: The Perspective of Neuroscience

“The heart asks pleasure first,

And then, excuse from pain;

And then, those little anodynes

That deaden suffering…”

Emily Dickinson, *Complete Poems*, 1862

Far from being a mere sensorial representation, pleasure can on the contrary constitute a complex psychic experience entailing various processes such as memory, motivation, homoeostasis, and, in some occurrences, pain. Furthermore, the hedonic marking of affects is the quality that, at a basic level, distinguishes emotions from other psychological processes (Damasio, 2004).

The complexity of affects, as phenomena behind the mechanisms of the brain regulating the development of painful or gratifying experiences, explains why, from a biological point of view, these were understood only partially until recent years; since then significant progress has been made by neuroscience in this field.

Pleasure is the subjective hedonic quality linked to stimuli or objects defined in behavioral terms as incentivizing or rewarding. The concept of reward, however, entails various neuropsychological components: first, the hedonic qualities linked to consumption (i.e., liking); second, the motivational/appetizing properties that drive an individual to obtainment (i.e., wanting); finally, the mnestic representation and the subsequent associative learning that derive from the achievement of these gratifying experiences (i.e., learning). Each of these components plays a key role in predisposing the biological resources in the brain that are necessary for evolutionary survival, guaranteeing an essential contribution to the success of adaptive behavior (Kringelbach and Berridge, 2010).

Analogously, the concept of pain entails both the hedonic aspect (i.e., suffering) and the motivational one (i.e., avoidance) of a painful experience. Clearly, the search for pleasure and avoidance of pain are important with regard to survival, and these two motivational elements compete with each other in the various mechanisms that regulate the functioning of the brain. A determining factor is subjective utility or individual motivation, termed *meaning*, which has been shown to be conditioned by sensorial, homoeostatic, and cultural characteristics (Leknes and Tracey, 2008). For instance, the motivational value of a stimulus increases if its effectiveness in restoring bodily homoeostasis is greater (Cabanac, 1979). This effect, known as *alliesthesia*, is particularly evident if we think of the incentivizing/hedonic properties of food, which increase when it has the function of alleviating hunger.

Because also painful experiences are a deviation from homoeostatic equilibrium, the same principle can be applied to pain and, in particular, to the pleasure deriving from the alleviation of pain. Thus, when a threat to the internal equilibrium of an organism increases, unpleasant sensations grow stronger, and defense and avoidance mechanisms are immediately activated (Leknes and Tracey, 2008).

Therefore, the alternating of pleasure and pain guarantees a constant optimization of our homoeostatic equilibrium. The influence of homoeostatic imbalance generated by hunger or thirst can be assessed in physiological terms, for instance, measuring glucose levels or blood volume, or from a behavioral point of view by looking at the increase of food and fluids consumption. However, research on animals has shown that the quantitative and qualitative characteristics of objects (i.e., incentivizing properties) can influence behavioral reactions and learning to a much higher degree than homoeostatic modifications (Mook, 1989). Thus, the decrease of the homoeostatic drive alone is not always effective (Panksepp and Biven, 2012).

Pleasure, therefore, cannot be defined simply as a sensation. Even the simplest sensorial pleasure, such as the one associated with something sweet, requires the contemporary involvement of other neuronal circuits aimed at adding a positive hedonic impact to the stimulus. Without this emotional nuance, even a feeling associated to something with a sweet taste may result as being neutral or even unpleasant (Kringelbach et al., 2012). Furthermore, the characteristics of pleasure are not only subjective but also objective. Although the subjective and conscious dimension associated to pleasure is the most evident, this dimension is underlain by objective neural systems that are selected and maintained in time by the same evolutionary metamorphoses that interest all the main psychological functions.

Hedonic experience requires the contemporary activation of neuronal circuits situated in mesocorticolimbic areas (Damasio, 2010; Panksepp, 2011; LeDoux, 2012) which have undergone an extraordinary evolution in time, precisely because affective reactions guarantee a significant objective gain for the organism (Darwin, 1872).

Biological systems of pleasure connote different experiences linked to the survival of the species in a positive hedonic sense, such as experiences deriving from relationships of attachment or sexual relationships, and have, for this reason, an adaptive function (Schore, 1994; Panksepp and Biven, 2012).

However, some central issues relating to the nature of pleasure, and more in general the nature of affects, are still object of debate in the field of neuroscience today. Among the most pressing issues: is it possible to hypothesize the presence of unconscious affects? Or, in other terms: is the origin of an affective experience to be located in cortical or in the subcortical region?

As observed by some authors, the subjective/conscious dimension of pleasure cannot be separated from the ancestral objective/unconscious dimension, linked to more simple subcortical circuits. The translation of stereotypical behavioral reactions, normally associated to hedonic experience, into more complex subjective and conscious sensations, however, requires the activation in human beings of additional cortical circuits specialized in the cognitive-experiential evaluation of stimuli (Berridge and Kringelbach, 2013). According to this approach, there is a marked difference between unconscious visceral-motor and behavioral manifestations associated with emotions, mediated by subcortical regions, and conscious affective experience, which is regulated by prefrontal cortex (PFC) and other cortical areas activity. Affects are thus constituted as a sort of cortical reading of physiological and automatic stimuli that are generated in subcortical region (LeDoux, 2002). On the contrary, other authors propose a radically different conceptual model, according to which the origin of affective perception can be located in subcortical brain regions, the activation of which supposedly influences a form of embryonic consciousness, defined *affective protoconsciousness* (Solms and Panksepp, 2012; Alcaro and Panksepp, 2014). This is described as being a form of consciousness centered around particular emotional states lacking an explicit objectual representation, a sort of “affective disposition” that is, however, necessary for the subsequent idiographic representation of experience (Northoff and Panksepp, 2008). Despite there being no differentiation between subject and object, this diffused state of affective consciousness is supposedly limited by an implicit sense of identity and differentiation that is established starting from the relationship between the perception of one’s own body (i.e., interoception) and that of the external environment (i.e., exteroception). Perception and regulation of interoceptive states is accompanied by affective states of pleasure or unpleasure according to whether the body state is of instinctive relaxation or tension or, in other words, depending on the degree of internal homoeostasis (Damasio and Carvalho, 2013). Accordingly, primordial exteroceptive sensations have an intrinsic affective connotation (Alcaro et al., 2017), as in the case of innate pleasure generated by a sweet taste or by the unpleasure caused by a bitter taste, and are always linked to the activation of motor sequences of active exploration mediated by emotional operating systems (Panksepp, 2010).

### Neurobiological Underpinnings of Pleasure: Brain Hedonic Systems

The sensation of pleasure linked to the consumption of tasty food differs from pleasure deriving from sexual intercourse or from pleasure deriving from substance abuse. Yet another different kind of pleasure is linked to experiences of socialization, or to the act of listening to music. Recent findings in the field of neuroscience have, however, demonstrated that a single functional circuit, incorporated inside the broader dopaminergic mesocorticolimbic system, seems to be involved in the various experiences of pleasure (Veldhuizen et al., 2010; Salimpoor et al., 2011; Georgiadis et al., 2012). Moreover, studies on animal models have recently identified a network for enhancing “liking” hedonic reactions, embedded as a set of small hedonic hot spots distributed among several limbic structures throughout the brain, ranging from the cortex to the brainstem (Berridge and Kringelbach, 2015). However, these hedonic hotspots are only partially overlapping with the so-called *brain reward system* (Berridge and Kringelbach, 2013), which was once thought to be at the origin of every sensation of pleasure, and that today some authors believe may mediate the enthusiastic drive to search and explore the environment in mammals (SEEKING System; Panksepp, 2010), while according to others its function is to mediate the expectation of gratification or, in a broader sense, to mediate desire (Berridge and Robinson, 1998).

Neuroimaging studies indicate that a distinct group of cortical [e.g., orbitofrontal cortex, anterior cingulate cortex (ACC), insular cortex] and subcortical regions (e.g., nucleus accumbens, amygdala, ventral pallidum) are activated by diverse hedonic stimuli in humans. Cortical hedonic representations (i.e., *encoding*) seem to be regulated by the activity of orbitofrontal cortex, particularly within medial and anterior regions (Murray et al., 2007). These structures seem to be particularly active in the subjective attribution of pleasure in reaction to a hedonic stimulus which may be of differing nature; also, they seem to mediate variations in the subjectively perceived hedonic intensity. Similarly, additional medial PFC areas, along with regions of the anterior insular cortex, seem to be connected to monitoring and anticipating pleasant objects reward value, as well as to the integration of perceptual stimuli with associated interoceptive states (Craig, 2009). However, it seems possible to trace the origin (*i.e., causation*) of affective experience, including hedonic experience, in subcortical regions more than in cortical ones, at least in humans. Cortical affective representation would thus imply elements that are inherent in cognitive contextualization of hedonic experience and second, the capacity of affective regulation and of decision-making. This aspect is demonstrated by the fact that relatively normal affective reactions continue to occur in human beings also when PFC and other cortical areas have been severely damaged (Damasio et al., 2013). The neural circuits believed to be responsible for the actual origin of hedonic experiences, at least of sensorial ones relating to the pleasure associated with sweet food, have been identified in brain stimulation experiments carried out on animals. In fact, the ability to experience pleasure in relation to sweet food is innate, just as expressive-facial manifestations associated with responses to these stimuli, which are, it must be noted, extremely evident in mammals (also in human new-borns; Berridge and Kringelbach, 2013). Thus, the hedonic impact of specific food can be measured objectively in rats by carefully observing their facial expressions, in particular the movements of their tongue (Steiner et al., 2001). From a neurochemical point of view, the neural systems implicated in the development of sensorial pleasure are much more limited than what was previously believed. For instance, it has been found that dopamine released in mesocorticolimbic region, in no way mediates any hedonic manifestation linked to consumption (i.e., *liking*), but rather it mediates aspects linked to motivation or, in a broader sense, to desire (i.e., *wanting*; Berridge, 2012). However, as some authors stress (Di Chiara, 2005; Panksepp, 2010), the release of dopamine within mesocorticolimbic regions may actually promote a behavioral state of appetite, intrinsically connected to positive affective states, even to hedonic ones (i.e., *state hedonia*; Di Chiara, 2005). Similarly, the neural centers responsible for the development of sensory pleasure are, from an anatomical point of view, much smaller than what was previously hypothesized, and that only the selective stimulation of μ opioid and endocannabinoid receptors located inside these centers can effectively amplify sensations of pleasure (Mahler et al., 2007; Smith et al., 2011). Specifically, opioid and endocannabinoid stimulation is able to amplify pleasure derived from consumption only in some specific sub-regions of the nucleus accumbens and of the ventral pallidum, while in other limbic structures, it only promotes an increase of appetitive motivation. From a functional point of view, NAc and ventral pallidum are deeply interconnected, so that the activity and integrity of both structures seems to be indispensable for maintaining normal hedonic reactions (Peciña and Berridge, 2005; Smith and Berridge, 2005).

### Interactions Between Pleasure and Pain

A large quantity of neuroscientific evidence indicates that there is a high degree of superimposition, in anatomical and functional terms, between areas of the brain and the neurotransmitter systems responsible for the regulation of physical pain and those responsible for the regulation of affective states. For instance, endogenous opioids and dopamine seem to be involved in a series of processes that take place in central and peripheral regions, among which the regulation of the motivational and hedonic aspects of reward, nociception, and modulation of physical pain and, in a broader sense, affective regulation (Leknes and Tracey, 2008). An increase of the activity of the μ and δ opioid receptors in the amygdala and in the ACC, other than being associated with deep analgesic states, seems also to be associated with a decrease of subjective unpleasantness experienced in response to nociceptive stimuli (Zubieta et al., 2001). On the contrary, a reduction of the activity of the μ receptors in the ACC region, of the amygdala and of the ventral pallidum, has been recorded during a prolonged recollection of painful memories, whereas an increase in the activity of the κ opioid receptor is generally associated with states of fatigue, confusion, dysphoria, and, at higher levels, with states of depersonalization (Zubieta et al., 2003a). In the region of the striate, the dopaminergic system seems to carry out various functions depending on the level of activation: states of tonic dopaminergic stimulation have been associated with an increase of an algesic response in relation to nociceptive stimuli, while phasic stimulation seems to have antinociceptive properties, perhaps involving the activation of μ opioid receptors (King et al., 2001; Zubieta et al., 2003b). The fine neurobiological interactions between dopaminergic neurotransmitter systems and opioids may finally explain some behaviors, such as self-harming behaviors, which are apparently conflicting with the principle which, according to some, guides human beings toward maximization of pleasure and avoidance of pain. As research on animals and humans has shown, self-harming components could be associated to a substantial release of endogenous opioids such as β-endorphines and enkephalins. Similarly, it has been demonstrated that algesic stimuli can reduce, through opioid receptor stimulation, the subjectively perceived intensity of emotional states connoted by a negative affective value. Finally, numerous studies have highlighted a reduction of basal levels of endogenous opioids in individuals who perform self-harming behavior or who are prone to suicide (Bresin and Gordon, 2013). From this point of view, according to some authors, self-harming behavior can be understood as the only way to regulate particularly painful, if nor traumatic, negative affective states, which cannot be otherwise symbolized (Fonagy et al., 2004; Stanley et al., 2010). Others believe that, because of the decreased basal levels of endogenous opioids, the release of β-endorphines, associated with the insurgence of self-harming acts, could have an effect of reward in these individuals (Bandelow et al., 2010). However, as is stressed by most authors, cumulative trauma can have an etiopathogenetic meaning in the genesis of self-harming behavior. Indeed, it seems that the repetition of traumatic episodes in early childhood can have, among its effects, a negative impact on the development of the young opioid and dopaminergic transmission systems (Schore, 1996).

## Pleasure and Affects: Psychoanalytic and Neuropsychoanalytic Developments

### Pleasure, Unpleasure, and Affect in Freud’s Theory

The issue of pleasure and, more in general, of motivation and affects, has a fundamental importance in the psychoanalytical theory because it is able to explain aspirations, thoughts, and behaviors of human beings from the point of view of subjective experience. Their role in psychoanalysis has been the object of debate for a century, a debate started with Freud’s formulation of the concept of drive, which is itself already a theory of motivation and affect (Yovell, 2008). Freud originally claimed that the guiding principle behind the functioning of human beings is the pleasure principle: that is the search for pleasure and avoidance of pain. The pleasure principle, as set out in the texts dating to 1900, is based on the idea that the psychic apparatus is constituted at the level of a reflex arc, the discharge of which is motility: the quantitative accumulation of excitement is indicated as unpleasure, its decrease as pleasure, while the fluctuation from one state to the other is desire, the only able to set the apparatus in motion, because the course of excitement is regulated automatically by the perception of the quantum of pleasure and unpleasure (Le Guen, 2008). Later, at the time of his writings on metapsychology, Freud fully elaborated what has been defined as the “drive model of the mind” (Greenberg and Mitchell, 1983). According to this model, man has, from birth, a motivational system, “pushes” that are on the border between the psychic and the somatic, an inner source of stimuli that influences and even guides the dynamics of the mind. The concept of drive is central in this model, “a request of work put forward to the mind,” a paraphysiological quantity of energy able to determine, from inside the psychic apparatus, the disturbance of a homoeostatic condition (Ammaniti and Dazzi, 1991).

Similarly, the aim of all drives is their satisfaction, achieved only through the suppression of the state of excitement that is present at the source. An affect, in this more complex explanation, is constituted again as the primary manifestation of a drive, equally elementary, and also grounded in biology, a qualitative and subjective expression of the quantity of drive energy. According to Freud: “an affect includes in the first place particular motor innervations or discharges and secondly certain feelings; the latter are of two kinds – perceptions of the motor actions that have occurred and the direct feelings of pleasure and unpleasure which, as we say, give the affect its keynote” (Freud, 1917, p. 395). Also: “we have decided to relate pleasure and unpleasure to the quantity of excitement that is present in the mind but is not in any way ‘bound’; and to relate them in a such a manner that unpleasure corresponds to an *increase* in the quantity of excitement and pleasure to a *diminution*.” (Freud, 1920, p.8)

Probably, Freud’s most original idea came from the fact that he intended affects as conscious perceptive modalities, experienced subjectively in the qualities of pleasure and unpleasure, and so relating to the Ego, whereas the unconscious affect is only a potentiality, blocked by the mechanism of repression. Previously, in his *Project for a Scientific Psychology* (1895), Freud had started to describe in theoretical terms the way experiences of pleasure and pain could interact with the issue of affects. In the chapter titled *The Experience of Satisfaction*, Freud puts forward his hypothesis on the economy of the mind: the endogenous psychic excitement cannot be arrested if not by a specific action brought on by an external object, an action a child, initially, is not capable of. And again: “in this way this path of discharge acquires a secondary function of the highest importance, that of communication, and the initial helplessness of human beings is the primal source of all moral motive” (Freud, 1895, p.318). From now on, pleasure will be associated with the image of the object that provided it and with the motor image of the movement of the reflex that allowed the discharge. Thus, according to Freud, affects are linked on the one hand to the function of communication, and so of language, and on the other to corporeal experience, by means of the motor image of discharge (Green, 1973).

### Neuropsychoanalytic Contributions to the Concept of Drive

The area of the brain in which the requests of the body are supposedly metalized could be located, according to some authors, in the hypothalamic region, and, more specifically, in the regions of the neural groupings specialized in the detection of homoeostatic physiological parameters and in the control of the activity of both the autonomic and the neuroendocrine nervous systems (Solms and Turnbull, 2002; Panksepp, 2010). The activity of these neural groups, both from an electrophysiological and neurochemical point of view, is able to evoke intense somatic, visceral sensations in human beings, at a subjective level, such as the ones linked to hunger, thirst, and sexual arousal. The Freudian concept of drive is deeply connected to energetic aspects, not only in terms of discharge, but also in terms of energy necessary to set the psychic apparatus in motion. As some authors have observed (Pfaff, 1999), such a mechanism predisposed to the generalized arousal of the whole activity of the brain can be found in all vertebrates, including human beings. This neural system, called BBURB (Bilateral, Bipolar, Universal Response Potentiating System), is thought to originate at the level of the brainstem’s medial and ventral reticular formations, which have projections to both superior and inferior anatomical areas. The ascending projections of the BBURB system are believed to enhance the sensorimotor response and the affective one in relation to stimuli that act with diverse modalities, while the descending ones enhance the autonomic response of the organism. The activity of the BBURB system thus guarantees the necessary quantity of energy for the promotion of all intrinsically motivated behaviors, from affective processes to cognitive ones, and, finally, also of the aspects linked to the emergence of individual consciousness. One of the branches of the BBURB system is thought to coincide with the ascending portions of the abovementioned mesocorticolimbic dopaminergic system (Panksepp, 1998). Thus, the latter constitutes the neuroanatomical substratum of what Panksepp (1998) has defined as the emotional SEEKING/desire system (SEEKING system). As some authors have observed in the field of neuropsychoanalysis (Solms and Turnbull, 2002; Yovell, 2008; Pfaff et al., 2014), the SEEKING emotional system displays a series of analogies with the Freudian concept of *libido*. The activity of the SEEKING system, in fact, promotes an appetitive predisposition in individuals, a euphoric mental state that is itself gratifying, which is thought to allow individuals to enter into relation with the surroundings in positive affective terms. This predisposition activates specific behavioral patterns (increased motor energy, exploration, and approach energy), and also affects the cognitive level, leading to associative reinforcement between gratifying experiences and the stimulus behind experience itself, through the creation of episodic memories. The activity of the SEEKING system thus predisposes the immediate organization of specific behavioral assets, which are thought to confer a direction to the action also when the object is not represented as the final goal (i.e., *intention-in-action*; Panksepp and Biven, 2012). This aspect characterizes the basic, unconditioned nature of the SEEKING system, in that the latter is able to unconditionally activate behavioral patterns (for instance, of search, approach, removal, attack), the aim of which becomes increasingly clear with the interaction taking place between the processes initiated by the basic affective systems (so at the level of subcortical regions) and neocortical areas. Thanks to these feedbacks between subcortical and neocortical areas, it is possible to construct patterns of relationship with the external object, with its potential ability to offer gratification and with our capacity to experience it by acting (Panksepp and Biven, 2012). The affective pre-representational (lacking an object) disposition, mediated by the activity of the SEEKING system, seems thus to project the organism toward external space, pushing it to act in a specific way. Only through interaction with the surroundings this affective disposition is, however, able to achieve full realization.

This characteristic inherent in the functioning of the SEEKING system can be seen in relation not only to the concept of *libido*, but also to some conceptual formulations by Bion and, in particular, to the notion of *pre-conception* (Bion, 1962). This is described by Bion as a sort of a priori knowledge, in psychoanalysis the equivalent of the Kantian concept of “empty thought,” its main quality being that it can be “thought” but not “become known.” In the mind of a newborn there is a preconception of the breast, an innate presentiment of it, which is, so to speak, “preformed.” What in fact characterizes preconceptions is essentially a sentiment of expectation that has the capacity to orient the newborn toward certain realization. Bion claims that when an expectation meets its corresponding realization, the psychological result is conception. In other words, when a newborn is breastfeeding, its preconception (or “idea”) of the breast is connected with its corresponding realization. Thus, conceptions (or “notions”) must be connected to an experience of satisfaction: not only at a physical level, but also at a cognitive one (Neri, 1987). Similarly, in the field of ethology, the theory of instinct says that the phenomenon of imprinting in a young animal is the encounter of a temporized predisposition and of an object present in its surrounding that realizes it, although imprinting seems to not entail forms of rewarding (Lorenz, 1988). Other than playing a fundamental role in the whole of the appetitive behaviors, the SEEKING system seems to be able to promote and energize dream activity (Solms and Turnbull, 2002). Also, the malfunctioning of this system seems to be behind some conditions such as mood disorders and pathological addictions (Zellner et al., 2011).

### Pleasure Beyond the Pleasure Principle: Motivations That Are Independent of Libido, Psychoanalytic, and Neuroscientific Contributions

A dilemma that Fred was not able to solve was the full understanding of what produces pleasure in human beings. Freud himself found that the hypothesis that pleasure is solely linked to the decrease of the drive tension was not completely satisfactory: it will suffice to think of the intensification of tension that is sought in sexuality (even though it later leads to an optimal decrease of tension). Similarly, the idea that all motivated behavior observable in human beings can be explained by the original libidic drive is not confirmed by evidence available today. Already in *Beyond the Pleasure Principle* (1920) Freud hypothesized that other drives coexist in the Ego beyond the libidic one and the self-preservation one, and that among these, a compulsion to repeat which is “more primitive, more elementary, more instinctual that the pleasure principle which it over-rides.” (Freud, 1920, p. 23) With regard to the genesis of this compulsion, according to Hartmann and the entire field of Ego psychology, Freud’s observations, relating to the existence of a primary motivation in human beings aimed at actively reproducing distressing events originally experienced passively, are significant: it is the case of separations, unexpected events, or traumatic experiences that are viewed as “having no explanation.” Hartmann (1939) writes: “the pleasure possibilities of the apparatuses of the conflict-free ego sphere seem, in any case, to play an important role in the adaptation to the external world, since the opening of such new sources of pleasure furthers ego development” (Hartmann, 1939, p.46). Here what Hartmann describes is pleasure for an activity, gratification in exerting a function that allows the Ego to “dominate reality,” as opposed to pleasure that originates from the mere satisfaction of a drive. It is from this principle that some later psychoanalytic concepts derive, such as competence (White, 1963), as well as the considerations by Rapaport (1953) relating to forms of human behavior, such as curiosity and the search for novelty, that are found to be direct expression of this principle. Motivation researchers in the field of psychology distinguish extrinsic motivation, based on the effect of attractive external rewards, from intrinsic motivation, which drives individuals into action regardless the incentive properties of environmental rewards, or internal homeostatic drives (Deci, 1971). Intrinsic motivation broadly refers to a set of psychological processes which includes the inherent propensity to pursue one’s choices, to seek out novelty and challenges, to satisfy curiosity and competence, and to extend one’s capacities and control over events. What these concepts have in common is an inner endorsement of one’s action, which is the sense that action is self-generated and is one’s own (Ryan et al., 1983).

Within this conceptual framework, the major distinction between intrinsic and extrinsic motivation has been postulated to be based on the degree of self-determination or, in a broader sense, on the degree of personal agency (Deci and Ryan, 1985). Agency refers to the experience of being in control of one’s own actions, and through them, to actively manage events in the external world. The neural basis of agency has generally been examined by comparing the neural activations of self-generated behavior to those of other-generated behavior (Lee and Reeve, 2013). Neuroimaging findings demonstrated that neural activities of the PFC, insular cortex, cerebellum and motor-related regions (e.g., supplementary motor area, pre-supplementary motor area, precentral gyrus, and postcentral gyrus) are related to the execution, observation, or imagination of self-generated behavior (Gallagher, 2000; Haggard, 2008; Nachev et al., 2008). Besides, the degree of agency is closely related to the degree of insular cortex activity, which provides a conscious representation of bodily self-related information (i.e., homestatic needs; Lee and Reeve, 2013). Research on the bodily self focuses on the development, maintenance, or disturbance of the link between a body and the experience of this body as “mine,” a process that is also known as body-ownership (Gallese and Sinigaglia, 2010). Body-ownership involves a complex neural network comprising the right temporo-parietal junction (which tests the incorporeability of the external object), the secondary somatosensory cortex (which maintains an on-line representation of the body), the posterior parietal and ventral premotor cortices (which code for the recalibration of the hand-centered coordinate systems), and the right posterior insula (which underpins the subjective experience of body-ownership; Tsakiris, 2010). Several approaches have attempted to explain the sense of agency and the sense of body-ownership: these two senses jointly constitute the sense of self and seem to derive from an interaction between current multisensory input and internal models of the body (Tsakiris, 2010). Regardless differences in the definition of the self, it is possible to identify “self-related processes” involving stimuli that are experienced as strongly related to one’s own person. The process of relating stimuli to the self should not be considered as an isolated phenomenon, but rather as embedded in a larger process depending on the environmental context (Salone et al., 2016). It has been suggested that 18-months-old infants who were coded as non-recognizers at the mirror-test task spent more time looking at the picture of their own face compared to the other-face, suggesting that before the onset of mirror self-recognition, featural information about the self might are more relevant in the process of recognizing one’s face, compared to multisensory cues (Filippetti and Tsakiris, 2018). Subortical-cortical midline structures (SCMS) are brain areas that enable self-related processing (SRP; Northoff and Panksepp, 2008). However, specific features of the self are also related to other cerebral regions (i.e., self-agency to right posterior insula, right inferior parietal cortex and motor-related areas, self-ownership to right parietal, ventromedial prefrontal, and insular cortices). The self therefore results from the integration of different brain regions, necessarily involving neural connectivity (Salone et al., 2016). A broader definition of SRP includes the coordination of various basic emotional processes and bodily interoceptive stimuli (e.g., emotional, motivational, homeostatic, bodily need states) with exteroceptive stimuli (e.g., sensory stimuli) in relation to the organism’s goal-directed activities (Northoff and Panksepp, 2008). Interestingly, a recent study demonstrated that SRP also induces neural activity in the same regions that are recruited by various rewards. This underlines that both reward processes and self-relatedness might share a similar evaluative process (de Greck et al., 2008). SCMS and their networks (e.g., right posterior insula, right inferior parietal cortex, ventromedial PFC) may represent neural correlates of the “core self,” defined as the continuous interaction between intero- and exteroceptive stimuli allowing the self to feel as a unit (Northoff, 2012). Within this conceptual framework, “core self” is thus to be considered as the subjective experience that oneself is the agent of perception, action, cognition, and emotion.

Some authors in the fields of psychoanalysis and biology have claimed that other proactive motivations that are independent of libido can be observed in human beings and in animals. One of these is no doubt attachment, today considered a primary motivation, biologically innate, which makes any attempt to explain it as a secondary element, with respect to the gratification of drives, awkward (Pine, 2005). To this regard, it must be noted that Freud elaborated two different theories of pleasure: a *quantitative* one, founded on the model of discharge-reduction of the drive tension, and a *qualitative* one, its model a type of sensual pleasure linked to child sexuality which is not easy to conceptualize in the terms set out by the principle of constancy (Eagle, 2013). According to Freud (1938): “the baby’s obstinate persistence in sucking gives evidence at an early stage of a need for satisfaction which, although it originates from and is stimulated by the taking of nourishment, nevertheless seeks to obtain pleasure independently of nourishment and for that reason may and should be described as *sexual*” (Freud, 1938, p. 154). Authors such as Fairbairn (1952) have claimed, in an even clearer way, that “libido is primarily object-seeking,” implying that object relations have an innate grounding, and implicitly refusing the centrality of drives in this context.

Later, the pioneering observation by Bowlby (1969) and, in a second moment, the review of literature on infancy by Stern (1985), made it clear once and for all that objectual attachment is present in human beings, in such an evident and precocious manner that it is possible to consider it in all respects as a primary and autonomous motivation.

Again, according to Stern (1990), the pleasure in children that can be observed during transitions characterized by secure attachment seems to be associated with moderate stimulation (and therefore excitement), rather than by a decrease or disappearance of excitement.

The work carried out by some authors in the field of neuroscience has allowed to identify with a higher degree of clarity the neural systems involved in attachment relationships in human beings and animals. One of the core motivational and emotional systems identified by Jaak Panksepp is CARE (Panksepp and Biven, 2012), which, other than being responsible for the promotion of attachment relationships, is also responsible for the creation of social bonds in a broader sense. Phylogenetically, this system could have evolved starting from other regions responsible for sexual desire and would thus share a certain neuroregulation with the latter.

From a neurochemical point of view, in fact, the high levels of oxytocin and endogenous opioids that can be observed in mothers caring for their offspring, could explain why the experience of caring for newborns is so gratifying for many mothers (Panksepp and Biven, 2012). Other authors have instead suggested that during periods that are critical for attachment the presence of stimuli associated with the mother’s face or tactile/auditory stimulation (Panksepp and Bishop, 1981; Schore, 1994) can induce gratifying affective states in the child, associated with the release of β-endorphins. Accordingly, endogenous opioid activity seems to play a role not only at the level of the tegmental dopaminergic mesolimbic system, promoting appetitive and approach behaviors toward the maternal object, but it also seems to play a role in the physiological development of the orbitofrontal system, a region characterized by a high density of endogenous opioids that is responsible for maintaining attachment patterns, beyond being responsible for the subsequent ability in affect regulation. Psychobiological attunement, interactive resonance, and the mutual synchronization and entrainment of physiological rhythms are fundamental processes that mediate attachment bond formation. Over the course of the first year after birth, limbic circuitries emerge in a sequential progression, from amygdala to anterior cingulate, to insula and, finally, to orbitofrontal cortex. As a result of attachment experiences, orbitofrontal cortex enters a critical period of maturation in the last quarter of the first year, the same time that working models of attachment are first measured (Schore, 2001). The orbital cortex matures in the middle of the second year, gradually allowing for an internal sense of security and self-regulation, which ultimately lead to the ability to regulate flexibly emotional states through interactions with other humans (Schore, 2001). The emergence of these flexible predictive capacities are dependent on extended parental investment and caring, through which the child becomes less rigidly controlled by the environment and more in tune with possibilities for action and gratification. It has been suggested that individual differences in the security of attachment and their sequelae can be viewed as reflecting, in part, variations in perceptions of personal agency among infants and toddlers (Ford and Thompson, 1985). Besides, various forms of attachment pathologies specifically represent inefficient patterns of organization of the right brain, especially the right orbitofrontal areas (Schore, 1994, 2001).

## To Conclude: Interactions of Integrations?

What has been discussed concerns, on the one hand, the complexity and, conversely, the risk of reductionism that connote the concept of pleasure, and on the other, the neurobiological substratum that supports the view of such complexity. Pleasure is not the mere absence of tension, a return to the central fluctuating state prescribed by homoeostasis: on the contrary, the use of substances and consequent addiction suggest that an allostatic model is better at predicting relapses and that the desire of a reward accompanies behaviors of sensation or novelty seeking (Pettorruso et al., 2014). Pleasure is inextricably linked to its “negative,” that is pain: the psychopathological and dynamic issue of masochism addressed by Freud (1924) in economic terms, reveals the drive interconnection of hedonic and anti-hedonic or destructive forces, which have potentially extreme consequences. Pleasure can become independent of libido and can include objects apparently unrelated to sexuality or instinctual satisfaction: perception of self-efficiency, cultural signification, and the propensity to communicate and form interpersonal relationships are telling examples of a type of “pleasure beyond the pleasure principle.” The concept of pleasure is closely connected to other bordering concepts, such as affect, desire, motivation, drive, which are not always unequivocally definable and differentiable and belong to different epistemological domains, such as experimental and cognitive psychology and psychoanalysis. What we have attempted to do, by necessarily restricting the field of research, is to describe the neural structures and neurochemical systems involved in the functioning of pleasure, without giving into the paradigm of simple localizationism. The advancement of neuroscientific knowledge actually allows to explain plurisemantic and sometimes paradoxical phenomena linked to pleasure and to the search of pleasure. The development of “bordering” or “bridging” disciplines, such as neuropsychoanalysis, that study conceptual methodologies of modeling of experimental data that can be understood with a theory of the functioning of the mind and that have possible clinical applications, seems to confirm the need for integrated knowledge (Moore et al., 2010; Leotti and Delgado, 2014; Leotti et al., 2015; Murayama et al., 2015). However, it is appropriate to ask whether the level of knowledge so far achieved is enough to speak of interaction, mutual influence, however, promising it may be, correspondences, or even of proper integrations. Writing about the border between psychoanalysis and neurosciences, already Modell (1996) remarked that “unification of ideas derived from neurobiology and psychoanalysis can help to illuminate a very broad and diverse range of problems extending from traumatic memories to the repetition compulsion, the psychoanalytic theory of instinct and the concept of the self.” Because both psychoanalysis and neurosciences have distinct and separate objects, methods, and types of knowledge, the objective of integration is complex and difficult to pursue in a clear way, that is, without running the risk of hyper-simplification or vagueness. Indeed, integration presupposes that two subjects, which are irreducible one to the other at a structural level, share or render compatible parts or functions of themselves. What parts or functions can psychoanalysis and neuroscience share? The issue forms the backdrop, as it were, or a challenge, for the emerging dialog.

The experience of pleasure, also beyond its limits and in the complexity of its interrelations, represents a useful and interesting testing site to attempt to integrate neuroscience and psychoanalysis. The change in psychobiological functioning that it entails, also in the long term, by means of the processes of learning and memory, evidently occurs as an only phenomenon that the insufficient instruments our knowledge depends on translates into two different and parallel orders of events, one that can be ascribed to the body (or the brain), the other to the mind. To leave the Cartesian dualism behind is the fundamental and ideal objective of this work in progress.

## Author Contributions

LJ contributed to article writing and personally revised and approved the final version of the manuscript. All authors revised and approved the final version of the manuscript.

## Conflict of Interest Statement

The authors declare that the research was conducted in the absence of any commercial or financial relationships that could be construed as a potential conflict of interest.
